# Stability of the frequent COPD exacerbator in the general population: A Danish nationwide register-based study

**DOI:** 10.1038/s41533-017-0029-7

**Published:** 2017-04-17

**Authors:** Mette Reilev, Jesper Lykkegaard, Anders Halling, Jørgen Vestbo, Jens Søndergaard, Anton Pottegård

**Affiliations:** 1 The Research Unit of General Practice, Department of Public Health, University of Southern Denmark, Odense, DK-5000 Denmark; 2 Department of Clinical Sciences Malmö, Center for Primary Health Care Research, Lund University, Lund, Sweden; 3 Department of Respiratory Medicine, Odense University Hospital, Odense, DK-5000 Denmark; 4 Division of Infection, Immunity and Respiratory Medicine, Manchester Academic Health Science Centre, University of Manchester and University Hospital South Manchester NHS Foundation Trust, Manchester, M23 9LT UK; 5 Clinical Pharmacology and Pharmacy, Department of Public Health, University of Southern Denmark, Odense, DK-5000 Denmark

## Abstract

Exacerbation frequency is central in treatment strategies for chronic obstructive pulmonary disease. However, whether chronic obstructive pulmonary disease patients from the general population with frequent exacerbations continue to have frequent exacerbations over an extended period of time is currently unknown. In this study, we aimed to investigate the stability of the frequent exacerbator in a population-based setting. To this end, we conducted a nationwide register-based descriptive study with a 10-year follow-up period of chronic obstructive pulmonary disease patients with at least one medically treated exacerbation in 2003. Each subsequent year, we divided the population into frequent, infrequent and non-exacerbators and quantified the flow between categories. Further, we estimated the percentage of frequent exacerbators at baseline who stayed in this category each year during a 5-year follow-up. We identified 19,752 patients with chronic obstructive pulmonary disease and an exacerbation in 2003. Thirty percent were frequent exacerbators. Overall, the majority of exacerbators in 2003 were non-exacerbators in the following years (60% in 2004 increasing to 68% in 2012). Approximately half of frequent exacerbators in one year experienced a decrease in exacerbation frequency and had either zero or one exacerbation in the subsequent year. This pattern was stable throughout follow-up. During a 5-year follow-up period, a substantial proportion (42%) of frequent exacerbators in 2003 had no additional years as frequent exacerbators, while the minority (6%) remained in this category each year. In conclusion, the rate of exacerbations shows considerable variation over time among chronic obstructive pulmonary disease patients in the general population. This might hold implications for chronic obstructive pulmonary disease treatment guidelines and their practical application.

## Chronic obstructive lung disease: Variations in disease progression

Patients with chronic obstructive pulmonary disease (COPD) who suffer from frequent exacerbations do not necessarily persist with such severity over time. Exacerbations in COPD are defined by worsening respiratory symptoms that result in changes to treatment, hospitalization and, at worst, death. However, clarity is needed on whether frequent exacerbations is a stable feature of some patients’ disease. Mette Reilev at the University of Southern Denmark and co-workers followed, over 10 years, 19,752 COPD patients living in Denmark who suffered at least one exacerbation in 2003. By 2004, 60% of patients were classed as infrequent or non-exacerbators, rising to 68% by 2012. Very few patients remained “frequent exacerbators”, suggesting the rate of exacerbations changes considerably over time. This could hold implications for COPD treatment and challenge assumptions made about disease progression.

## Introduction

Chronic obstructive pulmonary disease (COPD) is characterized by recurrent exacerbations, defined as episodes of “worsening of respiratory symptoms that are beyond the normal day-to-day variation and lead to a change in treatment”.^1^ Exacerbations have considerable impact on patients and result in increased mortality as well as augmented use of medication, rehabilitation and hospitalizations.^2–7^

Exacerbation frequency is a key component in determining treatment strategies for COPD. Most guidelines classify individuals by the number of exacerbations in the previous year, with the underlying assumption that frequent exacerbators remain in this category.^1^ To some extent, this assumption is supported by studies showing that the number of exacerbations in the previous year is the best predictor of future exacerbations.^8, 9^ In addition, the ECLIPSE study showed that the majority of patients without exacerbations remained stable over time, at least within the 3-year study period.^9, 10^ However, these studies included selected patients with moderate to very severe COPD in a hospital setting^11, 12^ who are known to differ substantially from COPD patients in primary care.^13^ Little is known about the stability of the exacerbation rate in the general COPD population over an extended period of time.

This study aimed at evaluating the stability of the frequent exacerbator status in the general COPD population in Denmark.

## Results

Among all Danish citizens who were 55 years or older (1.5 million), we identified 19,752 patients with at least one COPD exacerbation in 2003. The median age at cohort entry was 71 years (interquartile range 64–77), 57% were women, and 57% had experienced an exacerbation within the last 5 years (Table 1). 5917 patients (30%) were classified as frequent exacerbators and 13,835 (70%) as infrequent exacerbators; 50% of all exacerbators were classified as severe exacerbators. Compared to infrequent exacerbators, those classified as frequent exacerbators were more frequently previous exacerbators (75 vs. 49%) and used more respiratory drugs (Table 1).

**Table 1:** Baseline characteristics of the study population (31 December 2003)

	All (*n* = 19,752)	Infrequent exacerbators (*n* = 13,835)	Frequent exacerbators (*n* = 5917)	Severe exacerbators (*n* = 9853)
*Sex*				
Male	8452 (42.8%)	5848 (42.3%)	2604 (44.0%)	4379 (44.4%)
Female	11,300 (57.2%)	7987 (57.7%)	3313 (56.0%)	5474 (55.6%)
*Age (years)*				
Median (IQR)	71 (64–77)	70 (63–77)	71 (65–77)	72 (66–78)
55–69 years	8877 (44.9%)	6366 (46.0%)	2511 (42.4%)	3688 (37.4%)
70–79 years	7686 (38.9%)	5194 (37.5%)	2492 (42.1%)	4259 (43.2%)
80 + years	3189 (16.1%)	2275 (16.4%)	914 (15.4%)	1906 (19.3%)
*Previous exacerbations*				
No	8595 (43.5%)	7098 (51.3%)	1497 (25.3%)	3729 (37.8%)
Yes	11,157 (56.5%)	6737 (48.7%)	4420 (74.7%)	6124 (62.2%)
*Drug classes used*				
1	3574 (18.1%)	3058 (22.1%)	516 (8.7%)	1287 (13.1%)
2	5552 (28.1%)	4231 (30.6%)	1321 (22.3%)	2244 (22.8%)
3+	10,561 (53.5%)	6483 (46.9%)	4078 (68.9%)	6284 (63.8%)
*Medication*				
ICS	10,814 (54.7%)	7254 (52.4%)	3560 (60.2%)	5330 (54.1%)
LAMA	7007 (35.5%)	4306 (31.1%)	2701 (45.6%)	4286 (43.5%)
LABA	6512 (33.0%)	4181 (30.2%)	2331 (39.4%)	3572 (36.3%)
SABA	12,906 (65.3%)	8837 (63.9%)	4069 (68.8%)	6621 (67.2%)
SAMA	1254 (6.3%)	717 (5.2%)	537 (9.1%)	887 (9.0%)
Combination (SABA + SAMA)	8442 (42.7%)	5100 (36.9%)	3342 (56.5%)	5499 (55.8%)
Combination (LABA+ICS)	6940 (35.1%)	4484 (32.4%)	2456 (41.5%)	3663 (37.2%)

Individuals are classified according to rate and severity of exacerbations in year 2003

Non-exacerbators (0 exacerbations in the given year)

Infrequent exacerbators (1 exacerbation in the given year)

Frequent exacerbators (≥2 exacerbations in the given year)

Severe exacerbators (≥1 hospitalization with COPD in the given year)

*ICS* inhaled corticosteroids, *LAMA* long-acting muscarinic antagonists, *LABA* long-acting beta-agonists, *SAMA* short-acting muscarinic antagonists, *SABA* short-acting beta-agonists

**Fig. 1:**
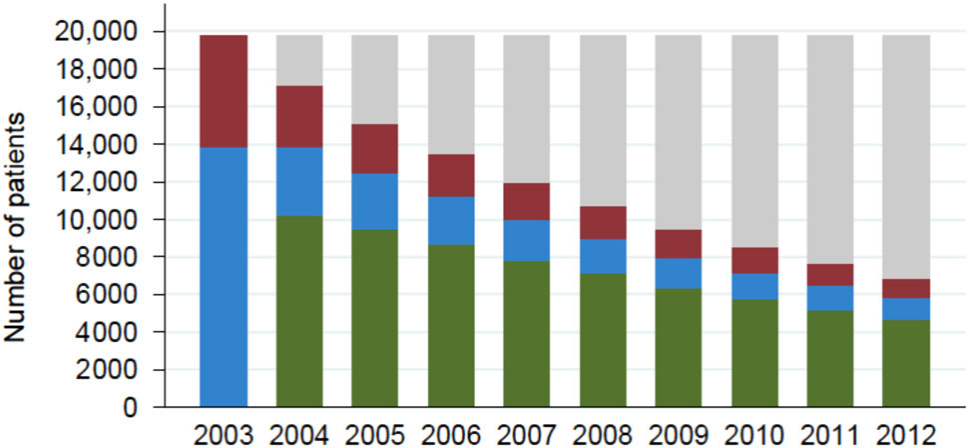
Distribution of exacerbators over time. Histogram 1 illustrates the number of frequent exacerbators, infrequent exacerbators and non-exacerbators each year among exacerbators at baseline (2003). *Red* Frequent exacerbators (≥2 exacerbations in the given year); *Blue* Infrequent exacerbators (1 exacerbation in the given year); *Green* Non-exacerbators (0 exacerbations in the given year); *Grey* Lost to follow-up (died or migrated)

As per inclusion criteria, all individuals had one or more exacerbations in 2003, of which 50% were classified as severe exacerbators. In 2004, 60% of those alive had no exacerbations and this proportion increased gradually to 68% in 2012 (Fig. 1). The proportion of exacerbators that were classified as severe exacerbators declined slightly throughout follow-up, to 43% in 2012.

The overall mortality among exacerbators in 2003 within the first year of follow-up was 13.2% (Fig. 1). The annual mortality rate was largely stable at around 11% during follow-up, with 65% having died by the end of 2012. Within the categories of non-exacerbators, infrequent exacerbators, frequent exacerbators and severe exacerbators, the annual mortality rate was likewise stable during the 10-year follow-up and was found to increase with increasing exacerbation rate (8, 13, 18, and 23%, respectively) (Table 2).

**Table 2:** One-year mortality according to exacerbation rate in the previous year

	All	Non-exacerbators	Infrequent exacerbators	Frequent exacerbators	Severe exacerbators
2004	13.2% (2606 of 19,752)		11.1% (1536 of 13,835)	18.1% (1070 of 5917)	19.0% (1873 of 9853)
2005	11.8% (2015 of 17,146)	8.9% (896 of 10,078)	13.0% (489 of 3760)	19.0% (630 of 3308)	23.0% (858 of 3736)
2006	11.0% (1662 of 15,131)	8.2% (768 of 9335)	12.6% (394 of 3139)	18.8% (500 of 2657)	22.7% (692 of 3046)
2007	11.1% (1499 of 13,469)	8.5% (729 of 8572)	13.7% (366 of 2670)	18.1% (404 of 2227)	23.4% (571 of 2441)
2008	10.6% (1267 of 11,970)	7.9% (613 of 7722)	13.1% (299 of 2279)	18.0% (355 of 1969)	23.8% (501 of 2105)
2009	11.1% (1188 of 10,703)	8.3% (591 of 7092)	13.4% (255 of 1898)	20.0% (342 of 1713)	25.0% (437 of 1748)
2010	10.4% (985 of 9515)	7.8% (491 of 6292)	13.1% (219 of 1673)	17.7% (275 of 1550)	22.7% (343 of 1513)
2011	9.9% (843 of 8530)	7.1% (408 of 5708)	13.8% (203 of 1476)	17.2% (232 of 1346)	23.0% (294 of 1276)
2012	10.2% (783 of 7687)	7.6% (390 of 5143)	12.6% (172 of 1370)	18.8% (221 of 1174)	24.8% (281 of 1132)

Non-exacerbators (0 exacerbation in the previous year)

Infrequent exacerbators (1 exacerbation in the previous year)

Frequent exacerbators (≥2 exacerbations in the previous year)

Severe exacerbators (≥1 hospitalization with COPD in the previous year)

**Fig. 2:**
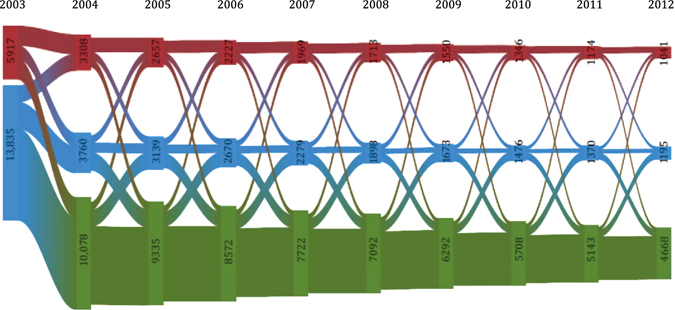
The stability at a population level over time. Riverplot illustrating the stability of the exacerbation rate over time among exacerbators at baseline. The size of the nodes is proportional to the percentage of individuals classified as frequent exacerbators, infrequent exacerbators and non-exacerbators, respectively. The thickness of the links between categories illustrates the size of the flow, i.e., the proportion of COPD patients within each category who are classified as either frequent exacerbators, infrequent exacerbators and non-exacerbators in the following year. The difference in volume between links and nodes represents the number of deaths each year. *Red* Frequent exacerbators (≥2 exacerbations in the given year); *Blue* Infrequent exacerbators (1 exacerbation in the given year); *Green* Non-exacerbators (0 exacerbations in the given year)

Throughout follow-up, about half of those classified as frequent exacerbators in 1 year, were classified as frequent exacerbators in the subsequent year (Fig. 2). Overall, a large proportion was classified as non-exacerbators from 2004 onwards (Fig. 2). The sensitivity analysis including the recommended first-line antibiotic treatment (amoxicillin combined with enzyme inhibitor) in the definition of exacerbations did not change the observed pattern (supplementary Fig. 1). When including all antibiotics used in the treatment of respiratory infections, a slightly larger proportion of those classified as frequent exacerbators in 1 year remained in this category in the subsequent year (supplementary Fig. 2). When restricting to individuals classified as frequent exacerbators at baseline, a largely similar pattern was observed (supplementary Fig. 3).

We performed a post-hoc analysis of exacerbators in 2003, restricted to those who were also classified as frequent exacerbators in 2002. In this population, a slightly larger proportion of those classified as frequent exacerbators in 1 year stayed in this category in the subsequent year, but the overall pattern was similar to that of the primary analysis (supplementary Fig. 4). Lastly, we performed a similar analysis, including mortality as a separate category (supplementary Fig. 5) which, in line with Table 2, illustrated that while the mortality was slightly higher among frequent exacerbators, the categories of infrequent exacerbators and non-exacerbators also contributed substantially to the mortality from 2004 and onwards.

The stability of frequent exacerbators at an individual level was assessed during a 5-year follow-up, restricting to 10,703 individuals (54%) that had five or more years of eligible follow-up. This restriction more often excluded those classified as severe exacerbators at baseline compared to frequent and infrequent exacerbators (61 vs. 55 vs. 42% excluded). Among those classified as frequent exacerbators in 2003, 5.6% (*n* = 150) remained in this category throughout the 5-year period while 16.4% (*n* = 437) experienced at least one exacerbation each year during the same period. The majority (84.6%, *n* = 2256) had at least 1 year with an exacerbation, however, almost half of those classified as frequent exacerbators at baseline (42.2%, *n* = 1124), were not classified as frequent exacerbators any other year throughout the 5 years of follow-up (Table 3). Stratification by age and sex showed no major differences, except a slight tendency towards fewer years classified as frequent exacerbators among individuals older than 80 years (data not shown).

**Table 3:** Stability at an individual level

Outcome	*N*	Percentage	Cumulative
*All exacerbators (entire cohort)*			
≥1 exacerbation annually			
for 5 out of 5 years	812	7.6%	7.6%
for 3 to 4 out of 5 years	2376	22.2%	29.8%
for 1 to 2 out of 5 years	4197	39.3%	69.0%
for 0 out of 5 years	3318	31.0%	100.0%
≥2 exacerbations annually			
for 5 out of 5 years	216	2.0%	2.0%
for 3 to 4 out of 5 years	907	8.5%	10.5%
for 1 to 2 out of 5 years	2901	27.1%	37.6%
for 0 out of 5 years	6679	62.4%	100.0%
*Frequent exacerbators at baseline*			
≥1 exacerbation annually			
for 5 out of 5 years	437	16.4%	16.4%
for 3 to 4 out of 5 years	875	32.8%	49.2%
for 1 to 2 out of 5 years	944	35.4%	84.6%
for 0 out of 5 years	410	15.4%	100.0%
≥2 exacerbations annually			
for 5 out of 5 years	150	5.6%	5.6%
for 3 to 4 out of 5 years	444	16.7%	22.3%
for 1 to 2 out of 5 years	948	35.6%	57.8%
for 0 out of 5 years	1124	42.2%	100.0%
*Severe exacerbators at baseline*			
≥1 exacerbation annually			
for 5 out of 5 years	364	9.5%	9.5%
for 3 to 4 out of 5 years	980	25.5%	35.0%
for 1 to 2 out of 5 years	1485	38.7%	73.7%
for 0 out of 5 years	1008	26.3%	100.0%
≥1 hospitalization annually			
for 5 out of 5 years	166	4.3%	4.3%
for 3 to 4 out of 5 years	612	15.9%	20.3%
for 1 to 2 out of 5 years	1469	38.3%	58.6%
for 0 out of 5 years	1590	41.4%	100.0%

The first section shows the proportion of individuals who experienced ≥1 exacerbation in year 2003 (i.e. all exacerbators) categorized according to the number of years in which they experienced ≥1 exacerbation or ≥2 exacerbations, respectively, throughout the first 5 years of follow-up. As an example, 8.5% of the study population, i.e., those who had an exacerbation in 2003, had ≥2 exacerbations in 3 or 4 years of the first 5 years of follow-up, while 62.4% had no years with ≥2 exacerbations within the same period. The table similarly shows the proportion of frequent and severe exacerbators in 2003, respectively, categorized according to the number of years in which they experienced ≥1 exacerbation, ≥2 exacerbations or ≥1 hospitalization throughout the 5 years of follow-up

Infrequent exacerbators (1 exacerbation in the given year)

Frequent exacerbators (≥2 exacerbations in the given year)

Severe exacerbators (≥1 hospitalization with COPD in the given year)

We conducted a similar analysis assessing the stability over a 3-year follow-up period, restricting to those with three or more years of eligible follow-up (*n* = 13,469, 68.2%). The overall pattern was generally similar to that of the 5-year analysis (supplementary table 1). Of those classified as frequent exacerbators at baseline, 47.9% (*n* = 1704) were not classified as frequent exacerbators in any of the subsequent 3 years, and 20.4% (*n* = 725) did not have any exacerbations at all.

## Discussion

### Main findings

In this population-based study covering the entire Danish population, we included all COPD patients experiencing an exacerbation in 2003 and followed them for up to 10 years. About half of those classified as frequent exacerbators in 1 year remained in this category in the following year, and an increasing proportion was classified as non-exacerbators throughout follow-up. At an individual level, the majority of the population experienced changes in the exacerbation frequency over time and very few remained frequent and severe exacerbators during a 5-year follow-up period.

### Interpretation of findings in relation to previously published work

To our knowledge, the stability of the frequent exacerbator over an extended period is only investigated in the ECLIPSE study. The ECLIPSE study found that the frequent exacerbator was relatively stable over a 2-year follow-up period, although the stability was more distinct among non-exacerbators.^9^ Contrary to our study, the ECLIPSE study was restricted to patients with moderate to very severe COPD treated in a specialist setting, and follow-up was limited to 3 years.^11^ Taking the differences in the study populations into account, the lack of a stable frequent exacerbator in the general population as indicated in this study is not necessarily conflicting with the results of the ECLIPSE study. A recent primary care study has estimated the tendency to remain in the same GOLD category over time,^14^ finding that the majority of patients in GOLD category D changed to less severe categories within 12 months of follow-up. This indicates variations in disease severity over time and as such, indirectly supports our findings.^14^

### Strengths and limitations of this study

The main strengths of our study is the large sample size and the nationwide approach with register data covering the entire nation, providing a unique opportunity to evaluate the stability of frequent exacerbators in a real-life setting over a long period through prospectively and independently collected register-based data from the general population.

According to previous studies, the COPD diagnosis has adequate completeness and a high positive predictive value (92%) in the Danish National Patient Register.^15^ Further, treatment with corticosteroids alone or in combination with antibiotics based on the Prescription Registry is considered a valid measure of COPD exacerbations in epidemiological studies.^16^ As such, identifying patients based on both an exacerbation and use of medication targeting obstructive pulmonary disease is considered to ensure a highly valid diagnosis of COPD in our study population.

The main limitation of the study was the lack of spirometry data, which prohibited a detailed assessment of disease severity. Thus, we cannot exclude that certain subpopulations of COPD patients might display a more stable disease trajectory. Similarly, some COPD patients and exacerbations might be misclassified as such when only based on registry data. To this end, we had excluded individuals <55 years and restricted to those experiencing an exacerbation requiring medical treatment in 2003. Further, our annual rate of exacerbations is comparable to a previous Danish population-based study.^17^ As such, we find it unlikely that a large proportion of individuals meeting these criteria do not have COPD and exacerbations. Further, our sensitivity analysis based on a population of patients who had exacerbations in both 2002 and 2003 led to similar conclusions. In addition, we could not assess to which extend prescriptions for oral corticosteroids were filled ahead of time, i.e., stockpiling. This would result in an overestimation of the exacerbation rate at first, and later, in an underestimation. We do not expect this to have a substantial impact on our results, as prophylactic fillings are rare in Denmark.

Another limitation of the study was that we were not able to account for the effect of inhaled bronchodilators and inhaled corticosteroids on the exacerbation rate. Long-acting muscarinic antagonist, long-acting beta-agonists, and inhaled corticosteroid-long-acting beta-agonists combinations have been shown to reduce the risk of exacerbations.^18–21^ A large proportion of the population received treatment with inhaled bronchodilators and/or inhaled corticosteroids at baseline, making it unlikely that a marked improvement in prognosis can be attributed to more extensive drug therapy. Further, the reported effect sizes are in general not assumed to explain frequent exacerbators to return to a non-exacerbator state and thus cannot explain the observed exacerbation rates in the present study.^18–21^

Defining exacerbations by the use of short-term oral corticosteroids induces a risk of misclassifying individuals as having exacerbations when using oral corticosteroids for other indications. However, the use of short-term oral corticosteroids for other indications than COPD is uncommon and would result in a slight overestimation of the rate of exacerbations. Hence, it would not affect our conclusion. Conversely, our definition of exacerbations based on use of oral corticosteroids does not capture untreated exacerbations as well as exacerbations treated with antibiotics only. As such, we underestimate the exacerbation rate but limit the outcome to moderate to severe exacerbations, which are expected to have the largest impact on the trajectory of COPD. On the other hand, including antibiotics in the definition of exacerbations, as in our sensitivity analyses, overestimates the exacerbation rate, as no antibiotics are exclusively labeled or used for exacerbations. However, it is of note, that even with the widest definition of exacerbations including all potentially relevant antibiotics, we observed large variations in the exacerbation rate (supplementary Fig. 2).

Healthy survivor effect is a potential bias in this study, as the mortality is higher among those classified as frequent and severe exacerbators (Table 2). The stability at an individual level could only be assessed in individuals who survived throughout the 3 or 5 years follow-up, which raises the question whether the skewed mortality infers a bias towards a less stable phenotype by selectively removing patients with frequent exacerbations. To this end, we note that a large proportion of the study cohort was classified as non-exacerbators or infrequent exacerbators also within the first years after inclusion where the cumulative mortality was limited. Consequently, a healthy survivor effect cannot in itself explain our results. While the cause of death is not known in our study, other studies have shown that primary causes of death in mild COPD are cardiovascular diseases and malignancies and, as COPD severity increases, respiratory diseases become a more frequent cause of death.^22, 23^ Among patients with moderate to very severe COPD, non-malignant respiratory diseases are the cause of death in about 35% of COPD patients.^24^ As such, we find it unlikely that the differences in mortality can explain our findings.

### Implications for future research, policy and practice

This study indicates that the trajectory of COPD in the general population is not necessarily progressive, at least with respect to exacerbation frequency. This might hold implications for treatment guidelines targeting COPD patients outside a specialist setting and for the practical application of these guidelines among patients with fluctuating disease severity. However, we acknowledge that our findings are controversial and consequently that they need to be confirmed in other populations and settings.

## Conclusions

In conclusion, this real-life study found considerable variations in the exacerbation rate over time and indicates that the majority of COPD patients in the general population do not persist with frequent exacerbations as a stable feature of their disease.

## Methods

We conducted a nationwide register-based epidemiological study with a 10-year follow-up period comprising all Danish patients with COPD who had at least one exacerbation in 2003.

### Data sources

Data were retrieved from Statistics Denmark, which is a governmental institution collecting electronic records for a broad spectrum of statistical and scientific purposes. The registries cover the entire Danish population of 5.4 million people (in 2003), of whom 1.5 million were 55 years or older, and contain detailed longitudinal register data at an individual level.^25, 26^ All Danish citizens are assigned with a unique civil registration number, which is used in all records and enables flawless linkage between registries.^26, 27^

The Danish National Patient Register contains administrative data on all hospital admissions since 1977 and contacts to outpatient clinics since 1995. The register contains information on date of admission and discharge diagnoses (ICD-10 since 1994).^28, 29^ The National Prescription Registry records information on medication for outpatient use sold on prescription since 1995.^30^ Each record includes date of purchase and the quantity dispensed. The dosing information and the indication for prescribing is not available as well as drugs dispensed at hospital level. Drugs are classified according to WHO’s anatomical-therapeutic-chemical (ATC) system and the quantity dispensed for each prescription is given by the number, strength and defined daily dose of the pharmaceutical entities. All medications targeting obstructive pulmonary disease (ATC R03) as well as corticosteroids (ATC H02) require a prescription.

### Population

We identified a closed cohort of individuals with at least one exacerbation requiring medical treatment from 1 January 2003 to 31 December 2003. We further required that individuals were ≥55 years and filled at least one prescription for medication targeting obstructive pulmonary disease (ATC R03) within 2003 (see Appendix 1). The composite requirement of high age, exacerbation status and the use of medication targeting obstructive pulmonary disease was applied to reduce the risk of including individuals with asthma in the COPD cohort. Further, the closed cohort of patients with exacerbations in 2003 allowed us to describe the trajectory of a patient population for 10 years. Inclusion and follow-up of one-year COPD exacerbators was estimated to account for roughly 5–7% of the total COPD population in Denmark.

### Definition of exacerbation rate

We defined a patient experiencing an exacerbation as a patient filling a prescription for a short-term course of oral corticosteroids (OCS) or being hospitalized due to COPD. Prescriptions of OCS and/or hospitalization had to be separated by 4 weeks to be considered separate events. This definition has previously been used in population-based studies^17, 31^ and is considered valid in an epidemiological setting.^15, 16^ A detailed definition of exacerbations is supplied in Appendix 2. Since indications for the use of medications are not available in the National Prescription Registry, we were unable to separate prescriptions targeted at exacerbations from prescriptions targeted at other infectious events. Therefore, exacerbations treated with antibiotics alone were not included in the primary analysis. Recognizing that exacerbations are sometimes treated with antibiotics alone, we performed two sensitivity analyses where we included prescriptions for antibiotics in the definition of exacerbations. First, we exclusively included prescriptions for amoxicillin and clavulanic acid (an enzyme inhibitor), as this is the recommended first-line antibiotic treatment of exacerbations in Denmark.^32^ Next, we included prescriptions for the most frequently used antibiotics in the treatment of exacerbations.^33^

According to the occurrence of exacerbations in a given calendar year, patients were classified as (1) non-exacerbators: 0 exacerbations, (2) infrequent exacerbators: 1 exacerbation, or (3) frequent exacerbators: ≥2 exacerbations. Further, we categorized patients with one or more hospitalizations due to COPD as severe exacerbators^31, 34^ Thus, severe exacerbators are either frequent or infrequent exacerbators.

### Analysis

To analyze baseline characteristics of exacerbators, we assessed registry data on age, sex and use of R03 medicine by the end of the inclusion period (31 December 2003). Individuals were classified as first time exacerbators or previous exacerbators, based on whether or not they had experienced at least one exacerbation in the 5 years prior to inclusion; i.e., in 1998–2002. To describe characteristics of the frequency of exacerbations and mortality over time, we determined the distribution of frequent exacerbators, infrequent exacerbators, non-exacerbators and individuals lost to follow-up each year from 2004 to 2012.

The stability of the frequent exacerbator phenotype was assessed using two different approaches. First, we classified exacerbators in 2003 as frequent exacerbators, infrequent exacerbators or non-exacerbators each year from 2004 to 2012, and estimated the flow between categories by calculating the annual proportion within each category that was classified as frequent exacerbators, infrequent exacerbators or non-exacerbators or was lost to follow-up (died or migrated) in the subsequent year. We performed a supplementary analysis restricting the population to individuals classified as frequent exacerbators in 2003.

As this analysis does not reveal the individuals’ tendency to remain in the same category more than 1 year, we secondly assessed the stability of frequent and severe exacerbators at an individual level during a 5-year follow-up period. To this end, we calculated the proportion of frequent exacerbators at baseline who remained classified as frequent exacerbators in 0, 1–2, 3–4 or 5 years or were classified as infrequent or frequent exacerbators in 0, 1–2, 3–4 or 5 years. Further, we calculated the proportion of individuals who remained classified as severe exacerbators for 0, 1–2, 3–4 or 5 years. This analysis was restricted to those eligible for follow-up throughout the entire 5-year period, and as such, the results from this analysis is sensitive to the considerable mortality among COPD patients. To test the influence of this restriction, we conducted a similar analysis using a 3-year follow-up period.

We performed supplementary analyses in which we stratified by sex and age (55–69, 70–79 and 80 + years at baseline). These categories represent birth cohorts with different exposures to risk factors for COPD (e.g., smoking and occupation) and potentially diverse trajectories of disease.

All analyses were performed using STATA 14.0 (StataCorp, College Station, TX, USA).

The study was approved by the Danish Data Protection Agency. According to Danish legislation, neither approval from the ethics committee nor informed consent from the study populations is required for registry-linkage studies.^28^

### Data availability

Analytical programs are available, while access to the dataset requires an application to The Danish Health Data Authorities.

## Electronic supplementary material

Supplementary information
