# Supplementary material for: Stability of the frequent COPD exacerbator in the general population: A Danish nationwide register-based study
Source: NPJ Prim Care Respir Med. 2017 Apr 17;27:25. doi: 10.1038/s41533-017-0029-7 (PMC5435093; doi:10.1038/s41533-017-0029-7)
Supplement: Supplementary file 1 — Supplementary information [file 41533_2017_29_MOESM1_ESM.docx]

**Supplementary information**

**Appendices**

**Appendix 1 – Definition and categorization of R03 drugs**

Medication targeting obstructive pulmonary disease was defined as drugs within the ATC-group R03. We excluded mono-therapeutic users of anti-allergic agents excl. corticosteroids (R03BC01 and R03BC03), inhaled epinephrine (R03AA01) or leukotriene receptor antagonists (R03DC). Mono-therapeutic use of these drugs was either negligible or not recommended in the treatment of COPD.

We classified drugs targeting obstructive pulmonary disease according to drug classes and calculated the proportion of users within each of the following categories: 1) short-acting β_2_-agonists (SABA), 2) long-acting β_2_-agonists (LABA), 3) short-acting anticholinergics (SAMA), 4) long-acting anticholinergics (LAMA), 5) inhaled corticosteroids (ICS), 6) combinations of short acting β_2_-agonists and short acting anticholinergics and finally 7) combinations of long acting β_2_-agonists and corticosteroids (**Table A1**). Combinations of long acting β_2_-agonists and long acting anticholinergics were not marketed until 2013 and therefore not included as a category.

**Table A1.** Most frequently used drugs targeting obstructive pulmonary disease and corresponding ATC-codes categorized according to effect. Only ATC-codes representing drugs that are allowed in Denmark and labelled COPD are included.

| **Drug** | **ATC codes** |
| --- | --- |
|  |  |
| *Short acting β_2_-agonists (SABA)* |  |
| salbutamol | R03AC02, R03CC02 |
| terbutalin | R03AC03, R03CC03 |
| fenoterol | R03AC04 |
| rimiterol | R03AC05 |
| orciprenaline | R03CB03, R03AB03 |
|  |  |
| *Long acting β_2_-agonists (LABA)* |  |
| salmeterol | R03AC12 |
| formoterol | R03AC13 |
| indacaterol | R03AC18 |
| olodaterol | R03AC19 |
| bambuterol | R03CC12 |
|  |  |
| *Short acting anticholinergics (SAMA)* |  |
| ipratropium bromide | R03BB01 |
| oxitropium bromide | R03BB02 |
|  |  |
| *Long acting anticholinergics (LAMA)* |  |
| tiotropium bromide | R03BB04 |
| aclidinium bromide | R03BB05 |
| glycopyrronium bromide | R03BB06 |
| umeclidinium bromide | R03BB07 |
|  |  |
| *Inhaled corticosteroids (ICS)* |  |
| glucocorticoids | R03BA |
|  |  |
| *Combinations (SABA+SAMA)* |  |
| fenoterol + ipratropium bromide | R03AL01 |
| salbutamol + ipratropium bromide | R03AL02 |
|  |  |
| *Combinations(LABA+ICS)* |  |
| salmeterol + fluticasone | R03AK06 |
| formoterol + budesonide | R03AK07 |
| formoterol + beclometasone | R03AK08 |
| vilanterol + fluticasone furoate | R03AK10 |
| formoterol + fluticasone | R03AK11 |

*ATC = Anatomical Therapeutic Chemical Classification System*

**Appendix 2 – Definition of exacerbation**

Short-term use of OCS was limited to fills of a maximum of 20 tablets of 25 mg in one day of either prednisolone (ATC H02AB06) or prednisone (H02AB07). Hospitalizations due to COPD were defined as admissions with a primary discharge diagnosis (ICD-10) of J40-44 (bronchitis, emphysema or COPD), or J96 (respiratory failure) or J13-18 (pneumonia) as the primary diagnosis combined with J41-44 as one of the secondary diagnoses. This definition of the COPD diagnosis was partly adopted from the Danish Register of Chronic Obstructive Pulmonary Disease.^1^ The COPD diagnosis has a high positive predictive value (92%) in the Danish National Patient Register.^2^

Prescription of OCS and/or hospitalization had to be separated by 4 weeks to be considered separate events.^3,4^

In sensitivity analyses we included prescriptions for amoxicillin and enzyme inhibitor (J01CR02) in the definition of exacerbations.^5^ Further, we made a sensitivity analysis where we included the most frequently used antibiotics, potentially indicated for exacerbations, i.e. amoxicillin and enzyme inhibitor (J01CR02), amoxicillin (J01CA04), pivampicillin (J01CA02), phenoxymethylpenicillin (J01CE02) and macrolides (J01FA01, J01FA06, J01FA09, J01FA10).^6^

**Supplementary results**

**Supplementary table 1.** The proportion of individuals in the entire cohort and among frequent and severe exacerbators classified as having any, frequent or severe exacerbations in 0, 1, 2 or 3 years throughout the 3 years of follow-up.

| **Outcome** | **N** | **Percentage** | **Cumulative** |
| --- | --- | --- | --- |
| *All exacerbators (entire cohort)* | | | |
| ≥ 1 exacerbation annually |  |  |  |
| for 3 out of 3 years | 2017 | 15.0% | 15.0% |
| for 2 out of 3 years | 2630 | 19.5% | 34.5% |
| for 1 out of 3 years | 3615 | 26.8% | 61.3% |
| for 0 out of 3 years | 5207 | 38.7% | 100.0% |
| ≥ 1 exacerbation annually |  |  |  |
| for 3 out of 3 years | 607 | 4.5% | 4.5% |
| for 2 out of 3 years | 1160 | 8.6% | 13.1% |
| for 1 out of 3 years | 2486 | 18.5% | 31.6% |
| for 0 out of 3 years | 9216 | 68.4% | 100.0% |
| *Frequent exacerbators at baseline* | | | |
| ≥ 2 exacerbation annually |  |  |  |
| for 3 out of 3 years | 1059 | 29.8% | 29.8% |
| for 2 out of 3 years | 881 | 24.8% | 54.5% |
| for 1 out of 3 years | 893 | 25.1% | 79.6% |
| for 0 out of 3 years | 725 | 20.4% | 100.0% |
| ≥ 2 exacerbation annually |  |  |  |
| for 3 out of 3 years | 402 | 11.3% | 11.3% |
| for 2 out of 3 years | 564 | 15.9% | 27.2% |
| for 1 out of 3 years | 888 | 25.0% | 52.1% |
| for 0 out of 3 years | 1704 | 47.9% | 100.0% |
| *Severe exacerbators at baseline* | | | |
| ≥ 1 exacerbation annually |  |  |  |
| for 3 out of 3 years | 997 | 18.0% | 18.0% |
| for 2 out of 3 years | 1196 | 21.6% | 39.7% |
| for 1 out of 3 years | 1522 | 27.5% | 67.2% |
| for 0 out of 3 years | 1813 | 32.8% | 100.0% |
| ≥ 1 hospitalization annually |  |  |  |
| for 3 out of 3 years | 576 | 10.4% | 10.4% |
| for 2 out of 3 years | 864 | 15.6% | 26.0% |
| for 1 out of 3 years | 1520 | 27.5% | 53.5% |
| for 0 out of 3 years | 2568 | 46.5% | 100.0% |

*Infrequent exacerbators (1 exacerbation in the given year)*

*Frequent exacerbators (≥2 exacerbations in the given year)*

*Severe exacerbators (≥1 hospitalization with COPD in the given year)*

**Supplementary figure 1.** Riverplot illustrating the stability of the exacerbation rate over time among exacerbators at baseline, when the definition of exacerbations include the recommended first-line antibiotic treatment for exacerbations (amoxicillin combined with enzyme inhibitor). The size of the nodes is proportional to the percentage of individuals with frequent exacerbators, infrequent exacerbators and non-exacerbators, respectively. The thickness of the links between categories illustrates the size of the flow, i.e. the proportion of COPD patients within each category that become either frequent exacerbators, infrequent exacerbators or non-exacerbators in the following year. The difference in volume between links and nodes represents the number of deaths each year. Red: frequent exacerbators (≥2 exacerbations in the given year); Blue: infrequent exacerbators (1 exacerbation in the given year); Green: non-exacerbators (0 exacerbations in the given year).

**Supplementary figure 2.** Riverplot illustrating the stability of the exacerbation rate over time among exacerbators at baseline, when the definition of exacerbations include all frequently used antibiotics, potentially indicated for exacerbations. The size of the nodes is proportional to the percentage of individuals with frequent exacerbators, infrequent exacerbators and non-exacerbators, respectively. The thickness of the links between categories illustrates the size of the flow, i.e. the proportion of COPD patients within each category that become either frequent exacerbators, infrequent exacerbators or non-exacerbators in the following year. The difference in volume between links and nodes represents the number of deaths each year.

Red: frequent exacerbators (≥2 exacerbations in the given year); Blue: infrequent exacerbators (1 exacerbation in the given year); Green: non-exacerbators (0 exacerbations in the given year).

**Supplementary figure 3.** Riverplot illustrating the stability of the exacerbation rate over time among those classified as frequent exacerbators at baseline. The size of the nodes is proportional to the percentage of individuals with frequent exacerbators, infrequent exacerbators and non-exacerbators, respectively. The thickness of the links between categories illustrates the size of the flow, i.e. the proportion of COPD patients within each category that become either frequent exacerbators, infrequent exacerbators or non-exacerbators in the following year. The difference in volume between links and nodes represents the number of deaths each year.

Red: frequent exacerbators (≥2 exacerbations in the given year); Blue: infrequent exacerbators (1 exacerbation in the given year); Green: non-exacerbators (0 exacerbations in the given year).

**Supplementary figure 4.** Riverplot illustrating the stability of the exacerbation rate over time among exacerbators in 2003 that were also classified as frequent exacerbators in 2002. The size of the nodes is proportional to the percentage of individuals with frequent exacerbators, infrequent exacerbators and non-exacerbators, respectively. The thickness of the links between categories illustrates the size of the flow, i.e. the proportion of COPD patients within each category that become either frequent exacerbators, infrequent exacerbators or non-exacerbators in the following year. The difference in volume between links and nodes represents the number of deaths each year.

Red: frequent exacerbators (≥2 exacerbations in the given year); Blue: infrequent exacerbators (1 exacerbation in the given year); Green: non-exacerbators (0 exacerbations in the given year).

**Supplementary figure 5.** Riverplot illustrating the stability of the exacerbation rate over time among exacerbators at baseline. The size of the nodes is proportional to the percentage of individuals who died or are classified as frequent exacerbators, infrequent exacerbators and non-exacerbators, respectively. The thickness of the links between categories illustrates the size of the flow, i.e. the proportion of COPD patients within each category who died or are classified as either frequent exacerbators, infrequent exacerbators and non-exacerbators in the following year.

Red: Frequent exacerbators (≥2 exacerbations in the given year); Blue: Infrequent exacerbators (1 exacerbation in the given year); Green: Non-exacerbators (0 exacerbations in the given year); Grey: cumulative proportion lost to follow-up, primarily due to death.

**References**

1. *Datadefinitioner.* Dansk register for Kronisk Obstruktiv Lungesygdom. DrKOL.;Marts 2015.

2. Thomsen RW, Lange P, Hellquist B, et al. Validity and underrecording of diagnosis of COPD in the Danish National Patient Registry. *Respiratory medicine.* 2011;105(7):1063-1068.

3. Ingebrigtsen TS, Marott JL, Lange P, Hallas J, Nordestgaard BG, Vestbo J. Medically treated exacerbations in COPD by GOLD 1-4: A valid, robust, and seemingly low-biased definition. *Respiratory medicine.* 2015.

4. Thomsen M, Ingebrigtsen TS, Marott JL, et al. Inflammatory biomarkers and exacerbations in chronic obstructive pulmonary disease. *JAMA : the journal of the American Medical Association.* 2013;309(22):2353-2361.

5. Hansen EFF, P; Titlestad, I. L.; Wessels, J. [Treatment guidelines for acute COPD exacerbations and non-invasive ventilation. Danish Society of Respiratory Medicine]; (in Danish). 2015.

6. Llor C, Bjerrum L, Munck A, et al. Predictors for antibiotic prescribing in patients with exacerbations of COPD in general practice. *Therapeutic advances in respiratory disease.* 2013;7(3):131-137.
